# Corrigendum: Absolute winding number differentiates mouse spatial navigation strategies with genetic risk for Alzheimer's disease

**DOI:** 10.3389/fnins.2022.1070425

**Published:** 2022-11-22

**Authors:** Alexandra Badea, Didong Li, Andrei R. Niculescu, Robert J. Anderson, Jacques A. Stout, Christina L. Williams, Carol A. Colton, Nobuyo Maeda, David B. Dunson

**Affiliations:** ^1^Department of Radiology, Duke University, Durham, NC, United States; ^2^Department of Neurology, Duke University, Durham, NC, United States; ^3^Brain Imaging and Analysis Center, Duke University, Durham, NC, United States; ^4^Biomedical Engineering, Duke University, Durham, NC, United States; ^5^Department of Computer Science, Princeton University, Princeton, NJ, United States; ^6^Department of Biostatistics, University of California, Los Angeles, Los Angeles, CA, United States; ^7^Department of Psychology and Neuroscience, Duke University, Durham, NC, United States; ^8^Department of Pathology and Laboratory Medicine, The University of North Carolina, Chapel Hill, Chapel Hill, NC, United States; ^9^Department of Statistical Science, Duke University, Durham, NC, United States

**Keywords:** APOE, Alzheimer's disease, brain, mouse, connectivity, memory, MRI

In the published article there was an error in the figures. Figure 2 was omitted, and Figure 3 was mistakenly used instead of Figure 2. The corrected Figure 2 appears below.

**Figure 2:**
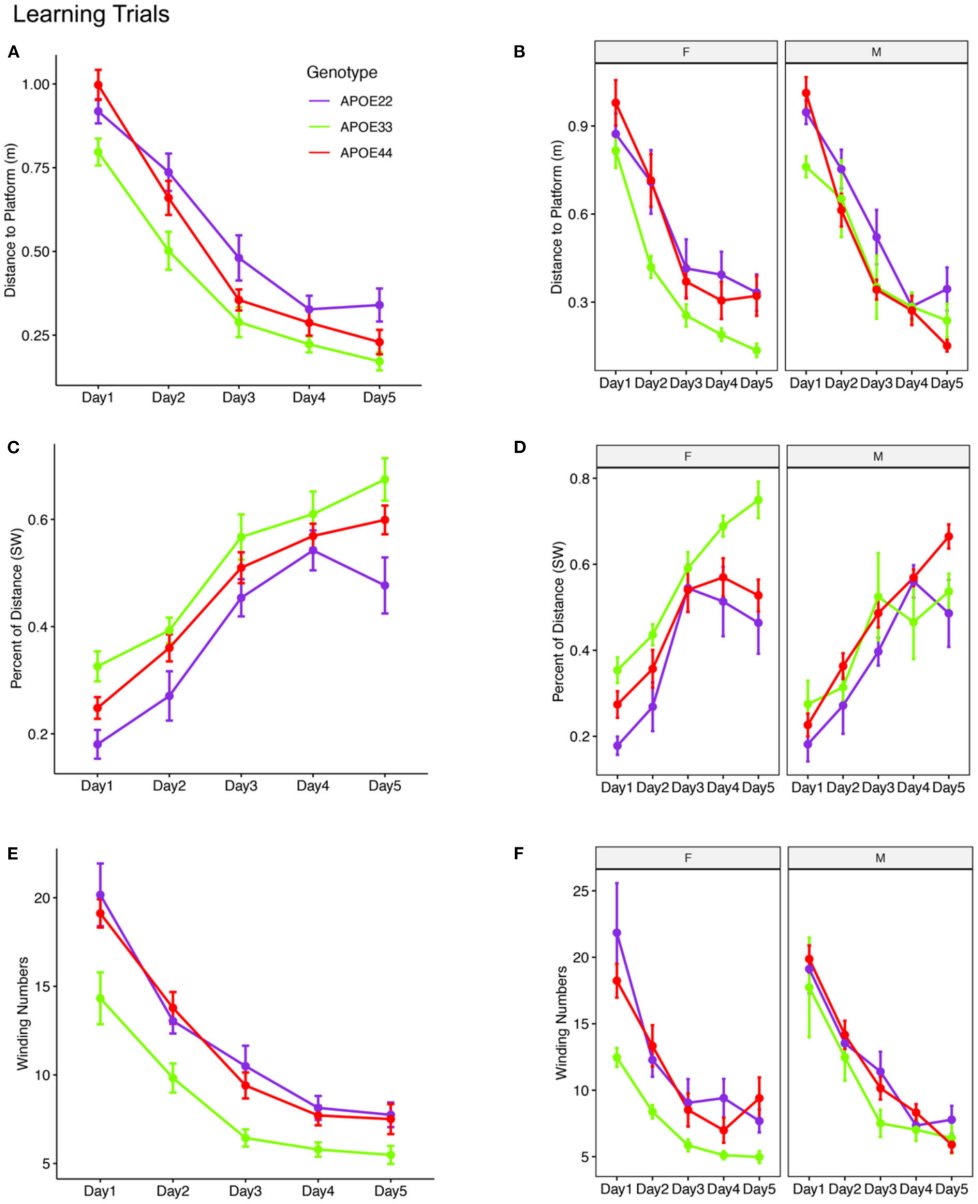
Learning trials. Mice swam shorter distances over the 5 testing days until reaching the hidden platform, indicating that they were learning **(A,B)**. Meanwhile, the percentage time swam in the target quadrant increased with time **(C,D)**. The absolute winding number clearly discriminated the APOE3 mice relative to APOE2 and APOE4 carriers, which used more similarly shaped trajectories **(E,F)**. The effects were larger in females across the 5 days. F, female; M, male. Graphs show mean ± standard error.

The authors apologize for this error and state that this does not change the scientific conclusions of the article in any way. The original article has been updated.

## Publisher's note

All claims expressed in this article are solely those of the authors and do not necessarily represent those of their affiliated organizations, or those of the publisher, the editors and the reviewers. Any product that may be evaluated in this article, or claim that may be made by its manufacturer, is not guaranteed or endorsed by the publisher.

