# Supplementary figures and images for: Absolute Winding Number Differentiates Mouse Spatial Navigation Strategies With Genetic Risk for Alzheimer’s Disease
Source: Front Neurosci. 2022 Jun 17;16:848654. doi: 10.3389/fnins.2022.848654 (PMC9247395; doi:10.3389/fnins.2022.848654)

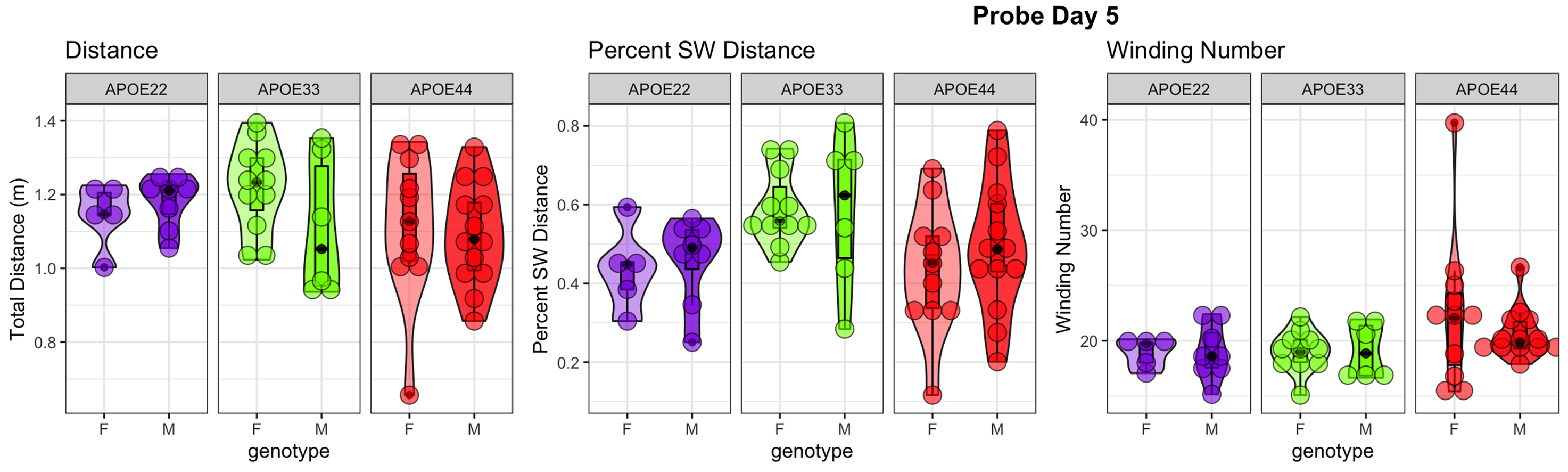

Supplement: Supplementary Figure 1 — Violin plots for the probe trials 1 h after ending the learning trials. Violin plots show median, interquartile (box), lower and upper adjacent data values, kernel probability density for the data. [file Image_1.PNG]

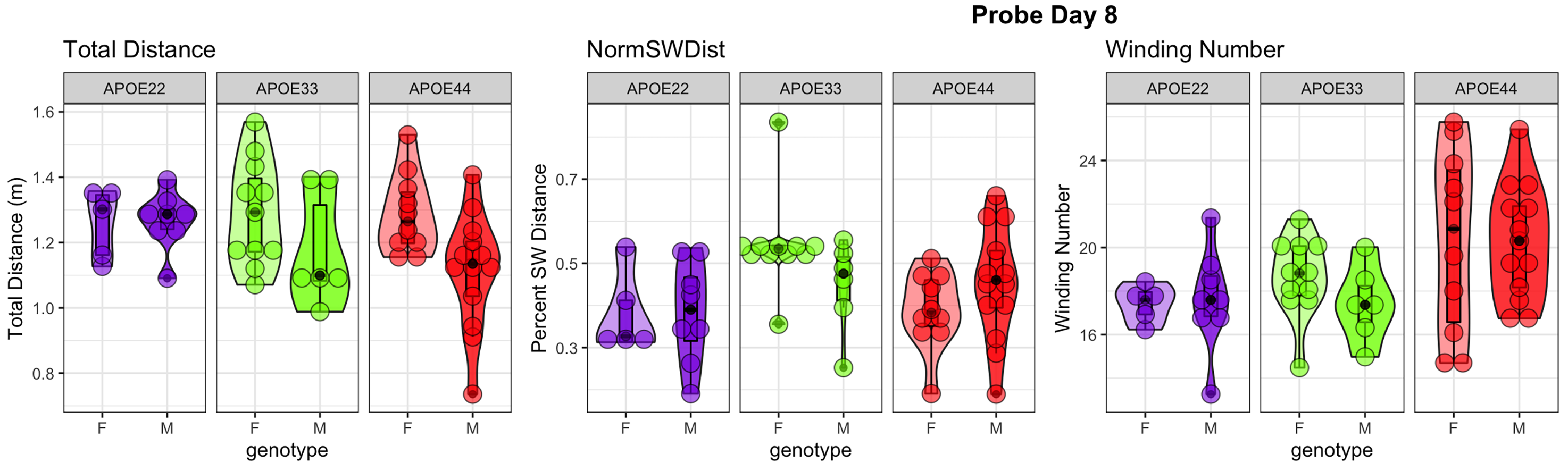

Supplement: Supplementary Figure 2 — Probe trials 3 days after ending the learning trials. Violin plots show median, interquartile (box), lower and upper adjacent data values, kernel probability density for the data. [file Image_2.PNG]

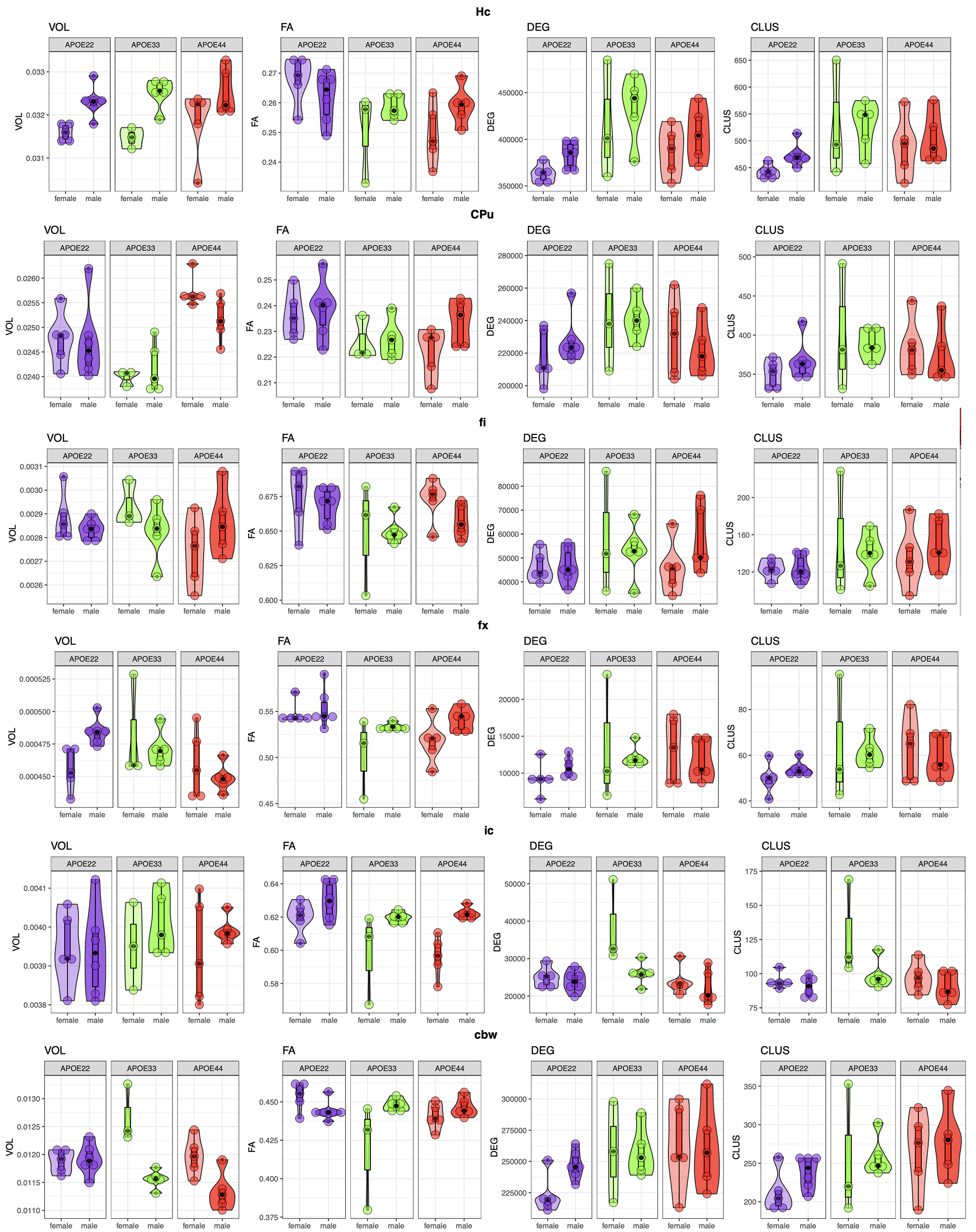

Supplement: Supplementary Figure 3 — Violin plots for imaging and network markers for volume, FA, degree of connectivity and clustering coefficient. [file Image_3.PNG]
