# Supplementary material for: Absolute Winding Number Differentiates Mouse Spatial Navigation Strategies With Genetic Risk for Alzheimer’s Disease
Source: Front Neurosci. 2022 Jun 17;16:848654. doi: 10.3389/fnins.2022.848654 (PMC9247395; doi:10.3389/fnins.2022.848654)
Supplement: Supplementary file 4 [file Data_Sheet_1.docx]

| Distance | SumSq | MeanSq | DF | NumDF | F | Pr(>F) | Eta_square | CI1 | CI2 | Cohen_f | CI1 | CI2 |
| --- | --- | --- | --- | --- | --- | --- | --- | --- | --- | --- | --- | --- |
| APOE | 0.52 | 0.26 | 2 | 48.00 | 8.44 | 0.001 | 0.26 | [0.06, | 0.44] | 0.59 | [0.26, | 0.88] |
| Sex | 0.02 | 0.02 | 1 | 48.00 | 0.50 | 0.485 | 0.01 | [0.00, | 0.13] | 0.10 | [0.00, | 0.38] |
| Stage | 14.38 | 3.60 | 4 | 192.00 | 117.16 | <2.20E-16 | **0.71** | [0.64, | 0.76] | 1.56 | [1.35, | 1.77] |
| APOE:Sex | 0.18 | 0.09 | 2 | 48.00 | 2.95 | 0.062 | 0.11 | [0.00, | 0.28] | 0.35 | [0.00, | 0.62] |
| APOE:Stage | 0.30 | 0.04 | 8 | 192.00 | 1.23 | 0.285 | 0.05 | [0.00, | 0.08] | 0.23 | [0.00, | 0.30] |
| Sex:Stage | 0.07 | 0.02 | 4 | 192.00 | 0.56 | 0.691 | 0.01 | [0.00, | 0.04] | 0.11 | [0.00, | 0.19] |
| APOE:Sex:Stage | 0.32 | 0.04 | 8 | 192.00 | 1.32 | 0.236 | 0.05 | [0.00, | 0.10] | 0.23 | [0.00, | 0.33] |
|  |  |  |  |  |  |  |  |  |  |  |  |  |
| NormSWDistance | SumSq | MeanSq | DF | NumDF | F | Pr(>F) | Eta_square | CI1 | CI2 | Cohen_f | CI1 | CI2 |
| APOE | 0.21 | 0.10 | 2 | 47.27 | 6.92 | 0.002 | 0.23 | [0.04, | 0.41] | 0.54 | [0.20, | 0.83] |
| Sex | 0.10 | 0.10 | 1 | 47.42 | 6.92 | 0.011 | **0.13** | [0.01, | 0.31] | 0.38 | [0.08, | 0.67] |
| Stage | 3.77 | 0.94 | 4 | 185.98 | 62.35 | <2.20E-16 | 0.57 | [0.48, | 0.64] | 1.16 | [0.96, | 1.34] |
| APOE:Sex | 0.21 | 0.11 | 2 | 47.27 | 7.00 | 0.002 | 0.23 | [0.04, | 0.41] | 0.54 | [0.20, | 0.83] |
| APOE:Stage | 0.09 | 0.01 | 8 | 185.76 | 0.77 | 0.634 | 0.03 | [0.00, | 0.05] | 0.18 | [0.00, | 0.23] |
| Sex:Stage | 0.03 | 0.01 | 4 | 185.98 | 0.54 | 0.708 | 0.01 | [0.00, | 0.04] | 0.11 | [0.00, | 0.19] |
| APOE:Sex:Stage | 0.24 | 0.03 | 8 | 185.76 | 1.98 | 0.051 | 0.08 | [0.00, | 0.13] | 0.29 | [0.00, | 0.38] |
|  |  |  |  |  |  |  |  |  |  |  |  |  |
| Winding | SumSq | MeanSq | DF | NumDF | F | Pr(>F) | Eta_square | CI1 | CI2 | Cohen_f | CI1 | CI2 |
| APOE | 185.70 | 92.86 | 2 | 48.00 | 9.10 | 0.000 | **0.27** | [0.07, | 0.45] | 0.62 | [0.28, | 0.91] |
| Sex | 25.40 | 25.43 | 1 | 48.00 | 2.49 | 0.121 | 0.05 | [0.00, | 0.21] | 0.23 | [0.00, | 0.51] |
| Stage | 4202.10 | 1050.52 | 4 | 192.00 | 102.95 | <2.20E-16 | 0.68 | [0.61, | 0.73] | 1.46 | [1.25, | 1.66] |
| APOE:Sex | 42.20 | 21.10 | 2 | 48.00 | 2.07 | 0.138 | 0.08 | [0.00, | 0.24] | 0.29 | [0.00, | 0.56] |
| APOE:Stage | 71.90 | 8.98 | 8 | 192.00 | 0.88 | 0.534 | 0.04 | [0.00, | 0.06] | 0.19 | [0.00, | 0.25] |
| Sex:Stage | 61.80 | 15.44 | 4 | 192.00 | 1.51 | 0.200 | 0.03 | [0.00, | 0.08] | 0.18 | [0.00, | 0.28] |
| APOE:Sex:Stage | 130.80 | 16.35 | 8 | 192.00 | 1.60 | 0.126 | 0.06 | [0.00, | 0.10] | 0.26 | [0.00, | 0.34] |

**Supplementary Table 1.** Statistical analyses for learning trials.

| DistanceDay5 | DF | SumSq | MeanSq | F | Pr(>F) | EtaSq | CI1 | CI2 | Cohen_f | CI1 | CI2 |
| --- | --- | --- | --- | --- | --- | --- | --- | --- | --- | --- | --- |
| APOE | 2 | 0.07 | 0.04 | 1.70 | 0.194 | 0.07 | [0.00, | 0.22] | 0.27 | [0.00, | 0.52] |
| Sex | 1 | 0.02 | 0.02 | 0.71 | 0.402 | 0.01 | [0.00, | 0.14] | 0.12 | [0.00, | 0.40] |
| APOE:Sex | 2 | 0.04 | 0.02 | 0.91 | 0.408 | 0.04 | [0.00, | 0.16] | **0.20** | [0.00, | 0.44] |
| Residuals | 48 | 1.02 | 0.02 |  |  |  |  |  |  |  |  |
|  |  |  |  |  |  |  |  |  |  |  |  |
| NormSWDistanceDay5 | DF | SumSq | MeanSq | F | Pr(>F) | EtaSq | CI1 | CI2 | Cohen_f | CI1 | CI2 |
| APOE | 2 | 0.19 | 0.09 | 4.48 | 0.016 | 0.16 | [0.01, | 0.33] | **0.43** | [0.07, | 0.71] |
| Sex | 1 | 0.01 | 0.01 | 0.47 | 0.495 | 0.01 | [0.00, | 0.13] | 0.10 | [0.00, | 0.38] |
| APOE:Sex | 2 | 0.01 | 0.00 | 0.21 | 0.808 | 0.01 | [0.00, | 0.09] | 0.09 | [0.00, | 0.31] |
| Residuals | 48 | 1.01 | 0.02 |  |  |  |  |  |  |  |  |
|  |  |  |  |  |  |  |  |  |  |  |  |
| WindingDay5 | DF | SumSq | MeanSq | F | Pr(>F) | EtaSq | CI1 | CI2 | Cohen_f | CI1 | CI2 |
| APOE | 2 | 80.96 | 40.48 | 3.02 | 0.058 | 0.11 | [0.00, | 0.28] | 0.35 | [0.00, | 0.62] |
| Sex | 1 | 9.45 | 9.45 | 0.71 | 0.405 | 0.01 | [0.00, | 0.14] | 0.12 | [0.00, | 0.40] |
| APOE:Sex | 2 | 12.88 | 6.44 | 0.48 | 0.621 | 0.02 | [0.00, | 0.12] | 0.14 | [0.00, | 0.38] |
| Residuals | 48 | 643.08 | 13.40 |  |  |  |  |  |  |  |  |
| **Supplementary Table 2**. Statistical analyses for probe trials 1 hour after ending the learning trials (Day 5). | | | | | | | | |  |  |  |
|  |  |  |  |  |  |  |  |  |  |  |  |
| DistanceDay8 | DF | SumSq | MeanSq | F | Pr(>F) | EtaSq | CI1 | CI2 | Cohen_f | CI1 | CI2 |
| APOE | 2 | 0.06 | 0.03 | 1.41 | 0.255 | 0.060 | [0.00, | 0.20] | 0.240 | [0.00, | 0.50] |
| Sex | 1 | 0.16 | 0.16 | 7.30 | 0.010 | **0.130** | [0.01, | 0.32] | **0.390** | [0.09, | 0.69] |
| APOE:Sex | 2 | 0.07 | 0.03 | 1.58 | 0.217 | 0.060 | [0.00, | 0.21] | 0.260 | [0.00, | 0.52] |
| Residuals | 47 | 1.01 | 0.02 |  |  |  |  |  |  |  |  |
|  |  |  |  |  |  |  |  |  |  |  |  |
| NormSWDistanceDay8 | DF | SumSq | MeanSq | F | Pr(>F) | EtaSq | CI1 | CI2 | Cohen_f | CI1 | CI2 |
| APOE | 2 | 0.13 | 0.07 | 4.99 | 0.011 | **0.180** | [0.01, | 0.35] | 0.460 | [0.11, | 0.74] |
| Sex | 1 | 0.00 | 0.00 | 0.00 | 0.961 | 0.000 | [0.00, | 0.02] | 0.007 | [0.00, | 0.14] |
| APOE:Sex | 2 | 0.06 | 0.03 | 2.26 | 0.116 | **0.090** | [0.00, | 0.25] | **0.310** | [0.00, | 0.58] |
| Residuals | 47 | 0.62 | 0.01 |  |  |  |  |  |  |  |  |
|  |  |  |  |  |  |  |  |  |  |  |  |
| WindingDay8 | DF | SumSq | MeanSq | F | Pr(>F) | EtaSq | CI1 | CI2 | Cohen_f | CI1 | CI2 |
| APOE | 2 | 75.76 | 37.88 | 5.29 | 0.008 | **0.180** | [0.02, | 0.36] | **0.470** | [0.13, | 0.76] |
| Sex | 1 | 1.35 | 1.35 | 0.19 | 0.666 | 0.004 | [0.00, | 0.11] | 0.060 | [0.00, | 0.34] |
| APOE:Sex | 2 | 4.57 | 2.29 | 0.32 | 0.728 | 0.010 | [0.00, | 0.11] | 0.120 | [0.00, | 0.35] |
| Residuals | 47 | 336.57 | 7.16 |  |  |  |  |  |  |  |  |

**Supplementary Table 3.** Statistical analyses for probe trials 3 days after ending the learning trials (Day 8).
